# Impact of romosozumab on serum calcium concentration and factors predicting the fluctuations in calcium concentration upon romosozumab administration: A multicenter retrospective study

**DOI:** 10.1016/j.bonr.2022.101635

**Published:** 2022-11-07

**Authors:** Hiroyuki Inose, Tsuyoshi Kato, Shoji Tomizawa, Akane Ariga, Takayuki Motoyoshi, Kazuyuki Fukushima, Kunihiko Takahashi, Toshitaka Yoshii, Atsushi Okawa

**Affiliations:** a Department of Orthopedic and Trauma Research, Tokyo Medical and Dental University, 1-5-45 Yushima, Bunkyo-ku, Tokyo 113-8519, Japan; b Department of Orthopaedics, Ome Municipal General Hospital, 4-16-5 Higashiome, Ome-shi, Tokyo 198-0042, Japan; c Department of Orthopedics, Tokyo Bay Urayasu Ichikawa Medical Center, 3-4-32 Todaijima, Urayasu-shi, Chiba 279-0001, Japan; d Department of Orthopedics, Saku Central Hospital Advanced Care Center, 3400-28, Nakagomi, Saku-shi, Nagano 384-0301, Japan; e Department of Biostatistics, M&D Data Science Center, Tokyo Medical and Dental University, 1-5-45 Yushima, Bunkyo-ku, Tokyo 113-8519, Japan; f Department of Orthopaedics, Graduate School, Tokyo Medical and Dental University, 1-5-45 Yushima, Bunkyo-ku, Tokyo 113-8519, Japan

**Keywords:** ALP, alkaline phosphatase, BMD, bone mineral density, DXA, dual-energy X-ray absorptiometry, P1NP, procollagen type 1 amino-terminal propeptide, SD, standard deviation, TRACP-5b, tartrate-resistant acid phosphatase 5b, Calcium, Osteoporosis, Bone density, Hypocalcemia

## Abstract

**Objectives:**

As romosozumab has both bone anabolic and antiresorptive effects, it is not clear which patient groups are more likely to have decreased calcium concentrations when treated with romosozumab. The aim of this study was to investigate the impact of romosozumab treatment on serum calcium concentration in patients with osteoporosis with a high risk of fractures and identify factors that might be associated with, or even predict, a fluctuation in calcium concentration upon romosozumab administration.

**Materials and methods:**

In total, 47 patients were included in this retrospective study. We performed a Wilcoxon signed-rank test to identify differences in the calcium concentration before and 1 month after romosozumab initiation. Associations between baseline variables and changes in serum calcium concentration were investigated with a multiple-linear regression model using a forward-backward stepwise procedure.

**Results:**

Romosozumab administration reduced the serum calcium concentration by an average of 3.1 % after 1 month. No patient complained of symptoms of hypocalcemia during the first month after treatment. Univariate regression analysis showed that age and calcium concentration were significantly associated with the decrease in serum calcium concentrations by romosozumab administration. In addition, stepwise regression analysis identified age and calcium concentrations as independent factors associated with the decrease in calcium concentration by romosozumab.

**Conclusion:**

Romosozumab administration caused a modest but significant decrease in serum calcium concentration. Older age and higher baseline calcium concentrations were associated with a greater decrease in calcium concentrations by romosozumab administration. Although the likelihood of severe hypocalcemia from romosozumab administration may be low, physicians prescribing romosozumab to patients with osteoporosis should be aware of the symptoms of hypocalcemia and promptly evaluate calcium levels if patients complain of these symptoms.

## 1 Introduction

With the aging of society, the prevalence of osteoporosis in adults is increasing (Vondracek and Linnebur, 2009). In March 2019, romosozumab, an anti-sclerostin antibody, was first approved in Japan for osteoporosis treatment in patients with a high risk of fracture. Romosozumab increases bone density by continuously promoting bone formation and inhibiting bone resorption early after administration (Cosman et al., 2016). A favorable increase in bone density with romosozumab use has been reported (Cosman et al., 2016; Ebina et al., 2021; Inose et al., 2022; McClung et al., 2014). However, despite vitamin D and calcium supplementations during clinical trials, hypocalcemia is considered an adverse effect of romosozumab administration (Cosman et al., 2016; McClung et al., 2014). In clinical practice, the administration of romosozumab causes a decrease in serum calcium concentrations throughout the administration period (Kobayakawa et al., 2021). The most common symptoms of hypocalcemia include paresthesia, muscle spasms, cramps, tetany, circumoral numbness, and seizures (Fong and Khan, 2012). Of note, acute hypocalcemia can cause severe symptoms requiring hospitalization, including laryngospasm, neuromuscular irritation, cognitive impairment, personality disorders, prolonged QT interval in electrocardiogram, changes mimicking myocardial infarction, and heart failure (Fong and Khan, 2012). In general, the use of antiresorptive drugs results in a decrease in calcium concentration due to the inhibition of calcium release from bones (Talreja, 2012). However, as romosozumab has both bone anabolic and antiresorptive effects, it is not clear which patient groups are more likely to have decreased calcium levels when treated with romosozumab. We hypothesized that if we could predict the degree of calcium reduction caused by romosozumab administration, we might be able to prevent the occurrence of hypocalcemia by taking measures such as checking the calcium concentration with blood tests and supplementing adequate levels of calcium and vitamin D. The aim of this study was to investigate the impact of romosozumab treatment for osteoporosis on serum calcium concentration in patients with a high risk of fractures and identify factors that predict the fluctuation in serum calcium concentration upon romosozumab administration.

## 2 Material and methods

### 2.1 Study population

We retrospectively enrolled 123 patients who received romosozumab for osteoporosis with a high risk of fractures in four hospitals (one academic medical center and three regional tertiary care hospitals) from March 2020 to March 2022. In this descriptive study, the inclusion criterion was having received romosozumab as a treatment for osteoporosis with a high risk of fractures. The following are the diagnostic criteria used by the Japanese Society of Bone Metabolism and the Japanese Osteoporosis Society to define osteoporosis with a high risk of fractures: 1) a bone mineral density (BMD) of ≤2.5 standard deviation (SD), with one or more fragility fractures; 2) lumbar vertebral BMD of <3.3 SD; 3) presence of two or more existing vertebral fractures; and 4) semi-quantitative evaluation results, indicating the presence of grade 3 vertebral fractures (Soen et al., 2013). Among the 123 enrolled patients, 76, whose serum calcium concentration was not measured after 1 month of romosozumab treatment, were excluded from the analysis. Patients with renal dysfunction (estimated glomerular filtration rate < 30 mL/min/1.73 m^2^) were not included in this study.

### 2.2 Ethics approval and informed consent

This study was approved by Medical Research Ethics Committee of Tokyo Medical and Dental University (approval number M2021–244) and internal review boards of the participating institutions, and conducted according to the Declaration of Helsinki recommendations. The opt-out method was used to obtain the patients' consent. Owing to the retrospective and anonymous nature of this study, the institutional review committee waived the requirement for informed consent from the patients.

### 2.3 BMD assessment

We performed dual-energy X-ray absorptiometry (DXA) to measure areal BMD at the spine (L1-L4 total), total hip, and femoral neck before and after 12 doses of romosozumab. Horizon (Hologic Inc., Bedford, MA, USA), Lunar iDXA (GE Healthcare Inc., Waukesha, WI, USA), and PRODIGY Fuga (GE Healthcare Inc.) were used as DXA equipment. The mean BMD percentage at the lumbar spine and femoral neck of young adults was reported by the Japanese Society of Bone and Mineral Research and Joint Review Committee of the Japanese Society for Osteoporosis, respectively, based on average values for adults aged 20–44 and 20–29 years (Soen et al., 2013). As this was a retrospective real-world study, vitamin D and calcium supplementations were not mandatory and were left to the treating physicians' discretion.

### 2.4 Serum measurements

Blood samples were collected before the first and second romosozumab administration. Blood samples were collected at the time of the outpatient visit and were not necessarily taken with the patients in the fasting state. As approximately 40 % of serum calcium is bound to albumin (Payne et al., 1973), we calculated albumin-corrected calcium concentration according to the formula provided in the K/DOQI guidelines:

$$\text{Albumin}-\text{corrected calcium concentration}=\text{total calcium}+0.8\times \left(4-\text{albumin}\right).$$

The correction was performed only when plasma albumin concentration was <3.5 g/dL (Gauci et al., 2008).

To evaluate the patient's bone metabolism, we measured bone turnover markers procollagen type 1 amino-terminal propeptide (P1NP) and serum total alkaline phosphatase (ALP) to assess bone formation and tartrate-resistant acid phosphatase 5b (TRACP-5b), a marker of osteoclast number, to indirectly evaluate bone resorption (Halleen et al., 2006; Inose et al., 2018).

We also analyzed other clinical factors [including age, sex, body mass index (BMI), as well as serum creatinine, albumin, and calcium concentrations], osteoporosis medications before romosozumab administration, and vitamin D and calcium supplementations. Information about previous osteoporosis treatments was obtained from medical records and outpatient questionnaires distributed to the patients.

### 2.5 Statistical analysis

After assessing data normality with the Shapiro-Wilk test, we performed a Wilcoxon signed-rank test to identify differences in the calcium concentration before and 1 month after romosozumab initiation.

The associations between the baseline variables and change in the calcium concentration were then investigated using a multiple linear regression model with a forward-backward stepwise procedure. First, the predictors associated with the dependent variable at *p* ≤ 0.25 in the univariate regression analysis were selected for inclusion in the model (Grant et al., 2019; Hosmer Jr. et al., 2013). Second, a stepwise model selection procedure was carried out among these candidates. Predictors with *p* > 0.1 were removed. The independent variables in the final model were controlled for multicollinearity. JMP version 14 (SAS Institute, Cary, NC, USA) was used for statistical analysis, and results with *p* < 0.05 were considered statistically significant. All data are presented as mean ± SD.

## 3 Results

In total, 47 patients were included in this study. Table 1 shows the baseline characteristics of these patients. Romosozumab administration caused a decrease of 3.1 % in the serum calcium concentration after 1 month (Table 2). The change in calcium concentration after 1 month of treatment with romosozumab ranged from +0.6 to −1.7 mg/dL. No patient complained of symptoms of hypocalcemia during the first month of treatment.

**Table 1:** Baseline characteristics of patients.

Characteristics	*N* = 47	Normal range
Age, years	77.8 ± 7.3	
Sex, n	Male 2 (4 %)	
BMI, kg/m^2^	20.7 ± 3.7	
Albumin, g/dL (*n* = 46)	4.0 ± 0.4	4.0 to 5.0
Creatinine, mg/dL (n = 47)	0.75 ± 0.2	Women 0.47 to 0.79 Men 0.61 to 1.04
ALP, U/L (*n* = 39)	280.0 ± 105.0	115 to 359
TRACP-5b, mU/dL (*n* = 41)	468.9 ± 200.4	Women 120 to 420 Men 170 to 590
P1NP, ng/mL (*n* = 34)	68.0 ± 43.6	Women 26.4 to 98.2 Men 18.1 to 74.1
Calcium, mg/dL (n = 47)	9.6 ± 0.6	8.5 to 10.2
Presence of prior anti-osteoporosis medication, n	22 (47 %)	
Details of osteoporosis treatment, n	Bisphosphonate 15 SERM 1 Teriparatide 5 Denosumab 1	
With vitamin D supplementation during romosozumab treatment, n	19 (40 %)	
Details of vitamin D/day, n	Eldecalcitol 0.75 μg 12 Eldecalcitol 0.5 μg 4 Alphacalcidol 0.5 μg 2 Unknown 1	
With Calcium supplementation during romosozumab treatment, n	2 (4 %)	
Mean percentage of YAM at lumbar spine, % (n = 47)	69.6 ± 13.1	
Mean percentage of YAM at femoral neck, % (n = 47)	58.3 ± 13.8	
Mean percentage of YAM at total femur, % (*n* = 43)	64.5 ± 13.7	

Data are presented as mean ± standard deviation or n (%).

BMI, body mass index; ALP, alkaline phosphatase; TRACP-5b, tartrate-resistant acid phosphatase 5b; P1NP, procollagen type 1 amino-terminal propeptide, SERM, Selective estrogen receptor modulator; YAM, young adult mean.

**Table 2:** Average calcium concentration before and 1 month after romosozumab administration.

Characteristic	Before	After 1 month	*P*
Calcium concentration	9.6 ± 0.6	9.3 ± 0.5	<0.0001⁎

Data are presented as mean ± standard deviation.

⁎ *P* < 0.05.

**Fig. 1:**
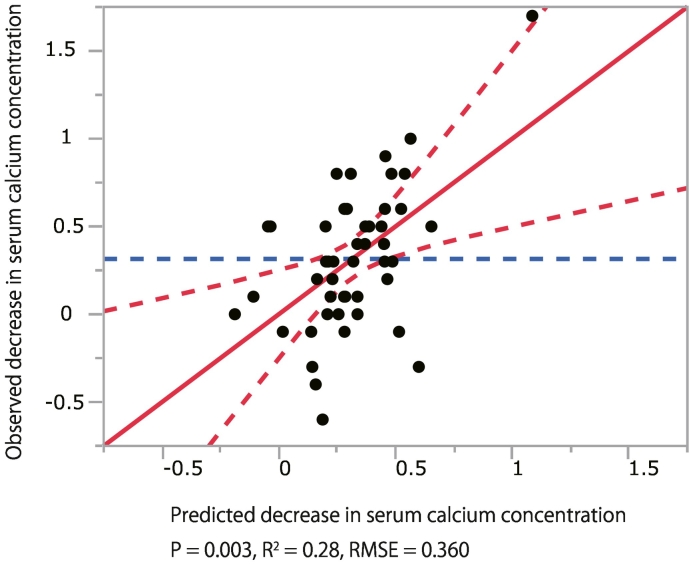
Observed versus predicted plots of the multiple linear regression model for the decrease in serum calcium concentration after romosozumab administration.

We performed a univariate regression analysis to identify factors associated with the decrease in calcium concentration. The results revealed that age and calcium concentration were associated with the decrease in calcium concentration (Table 3). Thereafter, independent predictors for the decrease in calcium concentration were investigated using the stepwise multiple regression analysis. Based on the univariate regression analysis, the dependent variable was defined as the decrease in calcium concentration, and the candidate independent variables were age; albumin; baseline calcium concentration; and baseline percentages of young adult mean at lumbar spine, femoral neck, and total femur. The stepwise regression analysis identified age and baseline calcium concentration as independent factors associated with the decrease in calcium concentration by romosozumab (Table 4 and Fig. 1). According to the predictive regression equation, a 1-year increase in age results in a 0.018-mg/dL (that is, a 10-year increase results in a 0.18-mg/dL) decrease in serum calcium concentration 1 month after romosozumab administration. Furthermore, a 1-mg/dL increase in serum calcium concentration before romosozumab administration results in a 0.249-mg/dL decrease in serum calcium concentration 1 month after romosozumab administration. Although not significant (*p* = 0.09), a 1 % increase in baseline percentage of young adult mean at lumbar spine results in a 0.007-mg/dL increase in serum calcium concentration 1 month after romosozumab administration.

**Table 3:** Univariate regression analysis. Association of baseline variables with the decrease in calcium concentration from before to after 1 month.

Characteristic	Estimation of partial regression coefficient	95 % CI	*P*	Standardized *β*
Age, years	0.019	0.003 to 0.035	0.02⁎	0.343
Sex	−0.018	−0.320 to 0.283	0.90	−0.018
BMI, kg/m^2^	0.015	−0.019 to 0.049	0.38	0.135
Albumin, g/dL	−0.250	−0.598 to −0.098	0.15	−0.213
Creatinine, mg/dL	−0.092	−0.904 to 0.720	0.76	−0.034
Calcium, mg/dL	0.289	0.084 to 0.495	0.007⁎	0.390
ALP, U/L	−0.0001	−0.001 to 0.001	0.84	−0.033
TRACP-5b, mU/dL	0.0001	−0.001 to 0.001	0.87	0.025
P1NP, ng/mL	−0.001	−0.005 to 0.002	0.35	−0.111
Presence of prior anti-osteoporosis medication, n	−0.056	−0.177 to 0.064	0.35	−0.140
With vitamin D supplementation, n	−0.008	−0.132 to 0.116	0.90	−0.020
With Calcium supplementation, n	−0.112	−0.411 to 0.187	0.45	−0.112
Baseline percentage of YAM at lumbar spine, %	−0.006	−0.015 to 0.004	0.23	−0.177
Baseline percentage of YAM at femoral neck, %	−0.008	−0.017 to 0.0003	0.06	−0.279
Baseline percentage of YAM at total femur, %	−0.008	−0.017 to 0.001	0.09	−0.265

ALP, alkaline phosphatase; BMI, body mass index; CI, confidence interval; P1NP, procollagen type 1 amino-terminal propeptide; TRACP-5b, tartrate-resistant acid phosphatase 5b; YAM young adult mean.

⁎ *P* < 0.05.

**Table 4:** Stepwise multiple regression analysis: independent predictors of the decrease in calcium concentration from before to after 1 month.

Factor	Estimation of partial regression coefficient	95 % CI	*P*	Standardized *β*	VIF
Calcium, mg/dL	0.249	0.052 to 0.446	0.01⁎	0.335	1.03
Age, years	0.018	0.003 to 0.033	0.02⁎	0.315	1.06
Baseline percentage of YAM at lumbar spine, %	−0.007	−0.015 to 0.001	0.09	−0.227	1.02

CI, confidence interval; VIF, Variance inflation factor; YAM, young adult mean.

⁎ *P* < 0.05.

## 4 Discussion

In the present study, we showed that romosozumab causes a decrease in serum calcium concentration and identified factors associated with this decrease. We found that 1 month after the first dose of romosozumab, the serum calcium concentrations decreased by 0.3 mg/dL (−3.1 %). In the Fracture Study in Postmenopausal Women with Osteoporosis (FRAME), the median serum calcium concentrations 1 month after romosozumab treatment were lower in the romosozumab group than in the placebo group (median change from baseline, −2.2 % vs. 0.0 %) (Cosman et al., 2016). Furthermore, in a study by Kobayakawa et al., there was a 2.5 % reduction after 1 month of romosozumab administration (Kobayakawa et al., 2021). Therefore, the extent of decrease in serum calcium concentration in this study is comparable to that observed in previous studies (Cosman et al., 2016; Kobayakawa et al., 2021). Although patients were supplemented with vitamin D_3_ or D_2_ and calcium in the FRAME study (Cosman et al., 2016), 40 % of the patients in our study were supplemented with an active vitamin D_3_ analog. According to Kobayakawa et al., patients receiving an active vitamin D_3_ analog presented significantly less calcium loss (Kobayakawa et al., 2021). In our study, according to the regression coefficient, although no significant differences were found, it could be considered that the administration of an active vitamin D_3_ analog has a protective effect against the reduction in serum calcium concentrations. Considering the inherent risks of developing hypocalcemia, it is safer to supplement patients with vitamin D after romosozumab administration.

Romosozumab reduced the serum calcium concentration after administration, but fortunately, no patient in our study developed the clinical symptoms of hypocalcemia. Hypocalcemia can range in severity from being asymptomatic in mild cases to presenting as an acute life-threatening crisis (Fong and Khan, 2012). Furthermore, the symptoms of mild hypocalcemia vary and lack specific subjective symptoms (Policepatil et al., 2012), suggesting that symptoms of mild hypocalcemia may often go undetected. In a prospective observational study, the highest decrease in the serum calcium concentration was 2.5 % up to 2 weeks after romosozumab administration, but the serum calcium concentration further decreased thereafter to 3.7 % at 12 months. In our study, one patient experienced a rapid reduction in calcium concentration (1.7 mg/dL) after receiving romosozumab. Although our study suggests that the likelihood of severe hypocalcemia from romosozumab administration is low, physicians prescribing romosozumab to patients with osteoporosis should be aware of the symptoms of hypocalcemia, such as paresthesia, muscle spasms, cramps, tetany, circumoral numbness, and seizures, and promptly evaluate calcium levels if patients complain of these symptoms.

Here, the stepwise multiple regression analysis revealed that age and baseline calcium concentration are independent factors associated with the decrease in serum calcium concentration by romosozumab. This implies that elderly patients with high baseline serum calcium concentrations are likely to experience a greater reduction in calcium concentration upon romosozumab administration. Although renal dysfunction is a risk factor associated with the development of hypocalcemia upon denosumab administration (Tsvetov et al., 2020), our results showed that the degree of reduction in calcium concentration upon romosozumab administration was not affected by renal function. In support of our findings, in a post-hoc study of clinical trials, the degree of decrease in serum calcium did not differ between patients with moderate renal dysfunction and those without renal dysfunction (Miller et al., 2022). However, it should be noted that in our study population, the creatinine concentrations were within the normal range in most patients; thus, we were not able to evaluate patients with impaired renal function.

This study has several limitations. First, it was a retrospective analysis, and the number of patients was rather small. Although there is no consensus on the appropriate sample size for multiple regression analysis, some studies have considered 10 events per variable reasonable (Harrell et al., 1996; Peduzzi et al., 1995). Based on this concept, the number of patients in this study was deemed sufficient given that there were >30 patients (10 patients per variable, for 3 variables in the final multiple regression model in this study). Additionally, the final prediction model had an R^2^ value of 0.28, indicating that it accounts for 28 % of the decrease in calcium concentration following 1 month of romosozumab treatment. According to the Cohen's guidelines, the fitted multiple regression model depends on R^2^, and if the value of R^2^ is between 0.02 and 0.12, the model is weak; between 0.13 and 0.25, it is moderate; and ≥0.26, it is good (Cohen, 1988). Accordingly, the fit of the prediction model was good. Therefore, we believe that the conclusions drawn are clinically useful, although external validation is needed in the future. Second, patients were not followed up for calcium concentration measurement over the entire period after romosozumab administration. However, because the greatest fluctuations in calcium concentrations after romosozumab administration reportedly occur during the first month (Kobayakawa et al., 2021), we focused on analyzing the changes in calcium concentration 1 month after romosozumab administration. Further prospective studies are required to address these limitations and confirm the results of this study.

## 5 Conclusion

Romosozumab administration caused a modest but significant decrease in serum calcium concentration. In addition, older age and higher baseline calcium concentrations were associated with a greater decrease in calcium concentration upon romosozumab administration. Although the likelihood of severe hypocalcemia from romosozumab administration may be low, physicians prescribing romosozumab to patients with osteoporosis should be aware of the symptoms of hypocalcemia and promptly evaluate calcium levels if patients complain of these symptoms.

## Funding

Not applicable.

## CRediT authorship contribution statement

**Hiroyuki Inose:** Conceptualization, Formal analysis, Investigation, Data curation, Writing – original draft, Visualization, Writing – review & editing. **Tsuyoshi Kato:** Investigation. **Shoji Tomizawa:** Investigation. **Akane Ariga:** Investigation. **Takayuki Motoyoshi:** Investigation. **Kazuyuki Fukushima:** Investigation. **Kunihiko Takahashi:** Formal analysis. **Toshitaka Yoshii:** Investigation. **Atsushi Okawa:** Supervision.

## Declaration of competing interest

The authors declare that they have no known competing financial interests or personal relationships that could have appeared to influence the work reported in this paper.

## Data Availability

The data that support the findings of this study are available from the corresponding author on reasonable request.
